# Manual Training in Schools

**Published:** 1890-10-11

**Authors:** 


### Manual Training in Schools.

### II.—IN AMERICA, SWEDEN, AND FRANCE.

In inquiring to what extent manual training has been recognised as part of the proper education of children, we naturally turn our attention first of all to kindergartens. The wisdom of Froebel's ideas on the subject of education is so generally recognised, and his schools for children of tender years are so well known, that we need not enter into any details here, and will only say that the very principle of a kindergarten is the education of children by manual training rather than by book-knowledge. They may almost be said to be at play while they are painting, stringing beads, or modelling in clay; yet, all unconsciously to themselves, they are being educated to habits of attention and of observation, as well as to really artistic manipulation, which will be of great service to them, not only in ordinary school-work, but in their after life.

Our American cousins have recognised the value of this kindergarten training more fully, or at least more practically, than we; for while we are content (or have until very lately been content) with private kindergartens here and there, they have gradually incorporated these schools into their public school system. In 1873 there were but 42 kindergarten schools in the United States; in 1880 there were 232; and by 1888 the number had risen to 521, giving instruction to over 31,000 children. There are also 55 training schools for kindergarten teachers; and it is reported on all hands that the results of the introduction of kindergarten teaching have been most satisfactory, not the least important of these results being that children of very tender years can now be received into the schools, instead of being left to run wild in the streets, and can be trained in habits of attention, order, &c., without any danger of forcing the development of their young minds.

There is obviously no reason why manual training should cease when the time arrives for the child to enter upon the usual course of school education; indeed, it is as important as ever it was. This the American School Boards have seen; and during the last six or eight years arrangements have been made in a large number of schools for lessons in various kinds of manual work. The cost of this new method of training is a serious consideration, special teachers being needed for some of these classes, as well as a certain amount of "plant"; but this is evidently not to be allowed to stand in the way of its success. In 1888 the Board of Education for New York appropriated fifteen thousand dollars to the manual-training department of twelve schools in the city; and there are now classes in these schools for carpentry work, modelling in clay, "construction work," paper and pasteboard work, industrial drawing, sewing, and cooking—history, arithmetic, and grammar receiving less attention than formerly, in order to make room for these new studies. As some of our readers may possibly be tempted to remark that cooking, however useful in after life, cannot be considered to have any educational value, we quote General Walker's remarks on the subject, taken from an article read to the National Educational Association:—

"No one can spend an hour in the cooking schools of Boston without being impressed by the very high educational value of the instruction given. As a great object lesson in chemistry; as a means of promoting care, patience, and forethought; as a study of cause and effect; as a medium of conveying useful information, irrespective altogether of the practical value of the art acquired, the course of lessons has constituted, I do not doubt, the best body of purely educational training any girl in all those classes ever experienced within the same number of hours."

We assent most heartily to this view of the matter; and we think that the art of killing two birds with one stone will never be more successfully practised than by those who introduce manual training into schools, giving mind-education and preparation for after life at one and the same time.

As the American boy can pass from the kindergarten to the manual training class, so he can pass from the manual training class to the Manual Training School. There are at least eighteen of these schools scattered over the States, some being supported by the municipalities, others by societies or by private individuals. Here the pupils receive instruction in drawing, wood-working, forging, machine-shop working, &c., the object of the schools being, as was said in our former article, to "teach the essential mechanical principles of all trades," and to provide a broad and liberal manual education. That the education there given is of the right sort may be seen by the testimony of the general foreman of a large system of railway shops:—

"As an employer, I will say for several of the manual school boys I have working for me that they will in one year accomplish as much as the ordinary boy will in three. One boy learns the trade by imitation, the other by reason and study. I give the manual school boy the preference, because I get much better results with less trouble."

As we are only concerned in this article with the course of manual training as a means of education, we will do no more than mention the Trade Schools of New York, Brooklyn, and other cities, in which boys and young men are taught one good mechanical trade, with the express purpose of enabling them to gain a livelihood.

### In Sweden.

It is interesting to compare the progress of manual training in America with that of Slöjd in Sweden, especially as the Boston Board of Education pronounces American and Swedish manners and institutions to be remarkably similar. In both countries manual training, while introduced in many schools, is not compulsory—indeed, in Sweden it is only given out of school hours to such children as volunteer to receive it; in both countries, again, it is advocated by two different parties, on two different grounds, one party valuing it for its influence upon the development of the pupil, the other for its money value. The director of the Näas Normal School for Slöjd instruction, however, emphatically declares that "whether the objects produced during instruction have a higher or lower market value, whether the children shall in future perform the same labour or not—these points of view are but incidental; not the products of labour, but the labourers themselves, are to be considered."

Slöjd instruction (the word Slöjd, by the way, is pronounced "Sloyd," and signifies manual dexterity—being, we imagine, a near relative of our word *sleight*) was introduced into Sweden in 1872, and of late years has attracted a good deal of attention, in our own country and elsewhere. We have not space to give any detailed description of this very interesting method of instruction, and can only say here that Slöjd includes carpentry, turning, and wood-carving, and that there is a regular set of thirty-three articles to be made by the scholar, beginning with a pointer and a flower-stick, and going on to a paperknife, a stocking-stretcher, a footstool, &c., &c., all the articles being arranged in the order of their difficulty. Each article must be made entirely by the pupil, and, when made, becomes his or her property.

### In France.

In 1882, manual training was introduced into the elementary schools of France, and so highly is it thought of, that it has now been made compulsory. It begins in the kindergartens, which are attended in large numbers by the children of the working classes, in age from two to six years. As these children pass on into the ordinary elementary classes, they are still given manual training, the boys in the higher classes learning wood and iron work, the girls household work and sewing. After leaving the elementary schools, French children can, if they choose, enter one of the *Ecoles Primaires Supérieures*, of which there are now seven in Paris alone. Here further training is given in wood and iron work, drawing, &c.; while in one of the schools girls are trained to be practical and intelligent workwomen, as well as to "take charge of the industrial establishments of woman's employment."

Besides these, there are the Apprentice Schools, the pupils in which belong mostly to the working classes. Here a course of instruction lasting three years is given, the first year being devoted to the study of various trades, the last two to that trade for which the pupil shows most aptitude. These French Apprentice Schools, therefore, would seem to be a sort of combination of the Manual Training Schools and the Trade Schools of America.

